# Influence of heat-assisted obturation on the distribution and post-setting stability of a calcium silicate–based endodontic sealer in simulated lateral canals: an in vitro study

**DOI:** 10.1186/s12903-026-09025-8

**Published:** 2026-07-03

**Authors:** Alberto Albero Monteagudo, Francesca Miola, María Grau Benítez, Nicolás Collado Castellanos, Angel del Campo-Rodríguez, Pedro Micó Muñoz

**Affiliations:** Department of Dentistry, Faculty of Health Sciences, Universidad Europea de Valencia, Valencia, Spain

**Keywords:** Bioceramic sealer, Lateral canals, Rroot canal filling, Warm obturation, Calcium silicate–based sealer, Thermoplastic obturation

## Abstract

**Background:**

To evaluate the effect of heat-assisted obturation on the distribution of filling material within apical and coronal lateral canals, the composition of the filling materials present within these canals, and the radiographic and microscopic characteristics of the premixed calcium silicate–based endodontic sealer after setting in standardized lateral canals.

**Methods:**

Sixty simulated root canals with standardized apical and coronal lateral canals (*n* = 120) were prepared in resin blocks. Canals were obturated using either a single-cone technique or carrier-based technique, both combined with a premixed calcium silicate–based sealer (CeraSeal^®^). Radiographic and microscopic analyses were performed immediately after obturation and after complete sealer setting (4 days, 37 °C, 100% humidity). Lateral canal filling was classified according to obturation completeness and material composition. Statistical analysis was performed using chi-square, Fisher’s exact, and McNemar tests (α = 0.05).

**Results:**

Heat-assisted obturation resulted in significantly higher rates of complete filling in apical lateral canals compared with the single-cone technique (90% vs. 46.7%, *p* < 0.001). Coronal lateral canals exhibited lower filling rates regardless of technique. Microscopic analysis showed that heat-assisted obturation promoted the combined presence of gutta-percha and sealer within lateral canals, whereas the single-cone technique resulted exclusively in sealer penetration (*p* < 0.001). No significant differences were observed between immediate and post-setting evaluations (*p* = 1.0).

**Conclusions:**

Heat-assisted obturation enhanced the distribution of filling materials within standardized lateral canals and promoted the concomitant presence of gutta-percha and sealer. No radiographic or microscopic differences were observed between the immediate and 4-day evaluations.

**Supplementary Information:**

The online version contains supplementary material available at https://doi.org/10.1186/s12903-026-09025-8.

## Introduction

The three-dimensional obturation of the root canal system is essential for the success of endodontic treatment, particularly due to the frequent presence of anatomical ramifications such as lateral canals [1, 2]. The persistence of pulp tissue or bacteria in these areas can sustain infectious processes and compromise the prognosis of the treatment [3, 4].

Clinical success depends on effective disinfection, complete debridement of the canal system, and a tight three-dimensional seal [3, 5]. However, anatomical variations such as isthmuses, ramifications, and accessory canals pose a clinical challenge, as they hinder proper cleaning and favor the persistence of bacteria [4, 6–8].

These canals may be located at three different levels within the root: coronal third, middle third and apical third. Approximately 73.5% oflateral canals are found in the apical third, in 11,4% in the middle third, and in 6,3% in the coronal third [9]. Accessory canals are rarely visible on preoperative two-dimensional (2D) radiographs; however, the presence of a lateral radiolucency may indicate existence [10].

To improve the three-dimensional adaptation of the obturation, various techniques have been developed, including warm vertical compaction and thermoplasticized gutta-percha carrier-based systems such as Thermafil^®^ [7, 11, 12]. In addition, the introduction of bioceramic sealers such as CeraSeal^®^ has provided bioactive properties, including biocompatibility, high sealing ability, and tissue regeneration promotion, representing a significant advancement over traditional materials [2, 3, 7, 13, 14]. CeraSeal is a premixed calcium silicate–based sealer mainly composed of calcium silicates, zirconium oxide, and thickening agents. Its fine particle size and hydrophilic properties may favor penetration into dentinal irregularities and lateral canals. Although bioceramic sealers like CeraSeal^®^ have demonstrated excellent sealing properties and bioactivity, there is limited evidence regarding their behavior when combined with thermoplastic techniques [3].

Previous studies have consistently demonstrated that thermoplasticized carrier-based techniques, such as Thermafil^®^, provide superior filling of lateral canals compared with cold obturation techniques. Classic studies by Reader et al. [15], Wolcott et al. [16], and DuLac et al. [17] reported a significantly greater capacity of warm gutta-percha systems to penetrate and fill lateral canals, whereas techniques such as single-cone or cold lateral condensation were primarily associated with sealer-only penetration.

However, most of these studies were conducted using conventional sealers, such as epoxy resin– or zinc oxide–based materials. Consequently, the behavior of contemporary premixed calcium silicate–based sealers when used in conjunction with heat-assisted obturation techniques remains incompletely understood.

In particular, the distribution of filling materials within standardized lateral canals and their radiographic and microscopic characteristics after setting have not been systematically evaluated.

Therefore, the present study aims to address this specific gap by evaluating the effect of heat-assisted obturation (Thermafil^®^) on the distribution, material composition, and post-setting behavior of a premixed calcium silicate–based sealer in a controlled experimental model. The null hypothesis was that heat-assisted obturation would not affect sealer distribution, material composition within lateral canals, or post-setting stability when compared with single-cone technique.

## Materials and methods

The study design was an in vitro experimental, descriptive, and analytical study. The research was carried out at the Universidad Europea Valencia. The manuscript was prepared in accordance with the 2021 Preferred Reporting Items for Laboratory Studies in Endodontology (PRILE) guidelines.

### Sample selection

Methacrylate structures containing main root canals with moderate curvature and two lateral canals—one coronal and one apical (Thermafil^®^ training block, Dentsply Sirona, York, PA, USA).)—were included. Resin blocks were selected to standardize canal anatomy and lateral canal location, reducing anatomical variability inherent to natural teeth. Structures lacking patency between the main canal and the lateral canals, whether due to manufacturer or operator error, were excluded.

### Sample size

A priori power analysis was performed to estimate the minimum sample size required to detect clinically relevant differences in complete obturation rates between groups. Considering expected proportions of 60% and 90%, a minimum of 60 simulated canals was considered sufficient to achieve 80% statistical power at a 95% confidence level.

### Procedure

#### Step 1: nomenclature, instrumentation, and patency verification

##### Nomenclature

Each acrylic platform was labeled with two metal numbers, one on each end. The canal to the left of the number was identified with the letter “A”, and the one to the right with the letter “B”.

##### Instrumentation

Size 10 K-files (25 mm) (Dentsply Sirona, Ballaigues, Switzerland) were used up to the apical constriction. The working length (18 mm) was calculated, and each canal was instrumented using the ProTaper Next^®^ rotary system (X1 and X2) (Dentsply Sirona, Ballaigues, Switzerland) at 350 rpm and 3.5 Ncms, following the manufacturer’s instructions. Water was used to irrigate between each file. Each file was used for the preparation of a maximum of ten canals.

During instrumentation, an X1 file fractured in canal A23, so an additional canal was prepared and obturated (Aextra1).

##### Patency verification

A syringe filled with water was used to verify the patency of each canal. First, the lateral canals were manually closed to check apical patency; then the apex was sealed to assess flow in the apical lateral canal; and finally, both the apex and the apical lateral canal were blocked to verify the patency of the coronal lateral canal. When no water flow was observed, a size 10 K-file was used.

#### Step 2: canals obturation

Thirty canals were obturated using the single gutta-percha cone technique and CeraSeal^®^ bioceramic sealer (Group 1), and another 30 using the Thermafil^®^ technique and CeraSeal^®^ bioceramic sealer (Group 2).

##### Group 1

ProTaper Next^®^ X2 Comfort Fit gutta-percha cones (Dentsply Sirona, Ballaigues, Switzerland) and CeraSeal^®^ bioceramic sealer (Meta Biomed Co., Ltd., Cheongju, South Korea) were used, applied with a 24-gauge needle to the middle third, following the manufacturer’s instructions. The gutta-percha point was inserted to the apical stop, the coronal end was cut with a heated instrument and compacted with a condenser.

##### Group 2

After drying the canal with paper points, CeraSeal^®^ bioceramic sealer (Meta Biomed Co., Ltd., Cheongju, South Korea) was applied with a 24-gauge needle to the middle third, following the manufacturer’s instructions. The Thermafil^®^ obturator (Dentsply Sirona, York, PA, USA) was heated in the Thermaprep 2^®^ (Dentsply Sirona, York, PA, USA) oven and inserted into the canal to the working length with continuous pressure. The carrier was removed using an Endo Access diamond bur (FG 1; Ref. A016430000100; 1.1-mm tip diameter; 21-mm length; Dentsply Sirona, Ballaigues, Switzerland).

#### Step 3: sealer setting

The samples were stored for 4 days in an incubator (HERATHERM IMC 18, Thermo Scientific^®^, Langenselbold, Germany) at 37 °C under 100% relative humidity, achieved by placing the specimens in a sealed humid chamber in accordance with the manufacturer’s recommendations for bioceramic sealer setting.

#### Step 4: data collection

Photographs were taken with a ZUMAX OMS 2380 microscope (16x) (Zumax Medical Co., Ltd., Suzhou, China) and radiographs with the VistaScan^®^ system (Dürr Dental SE, Bietigheim-Bissingen, Germany) (0.160s exposure) on the day of obturation (immediate control), and again 4 days later, using a LEICA DMS 1000 microscope (40x) (Leica Microsystems, Wetzlar, Germany). Two radiographs were taken per methacrylate plate. Each canal had four records: two photographs and two radiographs.

**Fig. 1:**
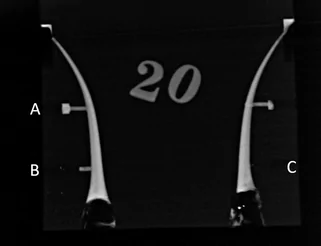
Representative radiographic images illustrating the classification criteria used for lateral canal obturation: (**A**) completely obturated canal, showing complete filling without radiolucent spaces; (**B**) partially obturated canal, characterized by incomplete filling of the lateral canal and/or the presence of radiolucent voids within the filling material; and (**C**) non-obturated canal, showing absence of filling material

**Fig. 2:**
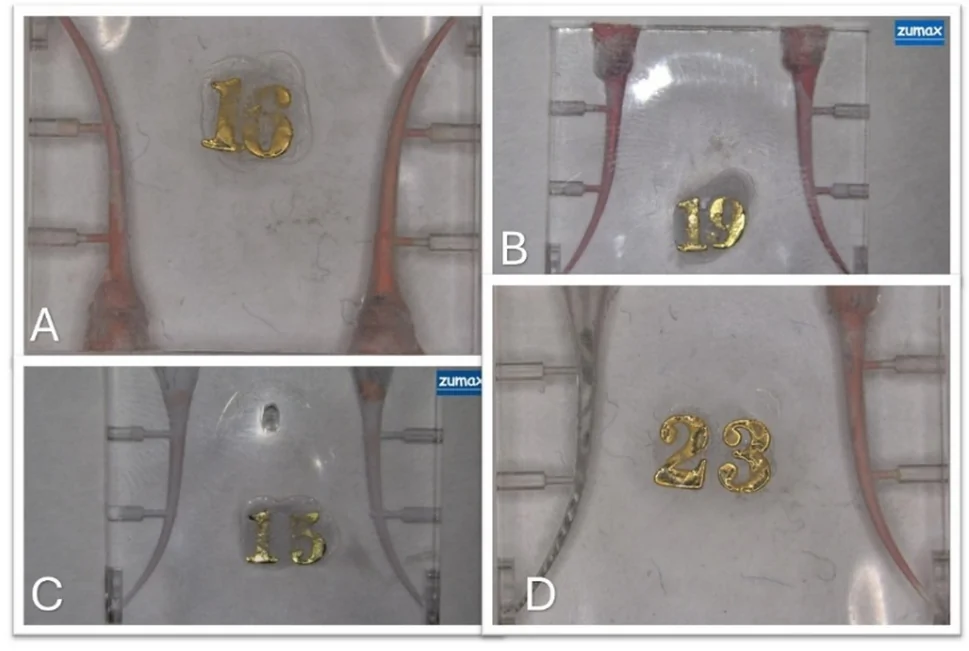
Representative microscopic images showing the type of obturation material within lateral canals: (**A**, **B**, **D**) combined gutta-percha and sealer penetration following the Thermafil^®^ technique; (**C**) sealer-only penetration following the single-cone technique. Images obtained at 16× magnification

Evaluation of results (Figs. 1 and 2).

Two calibrated examiners independently evaluated the radiographic and microscopic images. Prior to the final assessment, examiner calibration was conducted using representative images. Inter-examiner agreement was assessed using Cohen’s Kappa coefficient, which showed a high level of agreement (κ = 0.85). In cases of disagreement, a consensus was reached through joint evaluation.

#### Quantitative analysis

Radiographic classification of lateral canal obturation (Fig. 1):

- Completely obturated (complete filling of the lateral canal without radiolucent spaces.).
- Partially obturated (incomplete filling of the lateral canal and/or presence of radiolucent voids within the filling material.).
- Not obturated (absence of filling material within the lateral canal).
- Qualitative analysis:
- Photographic classification (Fig. 2):
- Gutta-percha only.
- Sealer only.
- Gutta-percha and sealer.

### Statistical analysis

Bivariate analyses were performed to assess the association between obturation technique and treatment outcome. Comparisons between techniques were conducted using the chi-square test or Fisher’s exact test when appropriate. Paired comparisons according to canal location (coronal vs. apical) and evaluation time were performed using McNemar’s test. Statistical significance was set at α = 0.05. Statistical analyses were performed using IBM SPSS Statistics (IBM Corp., Armonk, NY, USA) and R software (R Foundation for Statistical Computing, Vienna, Austria).

## Results

**Fig. 3:**
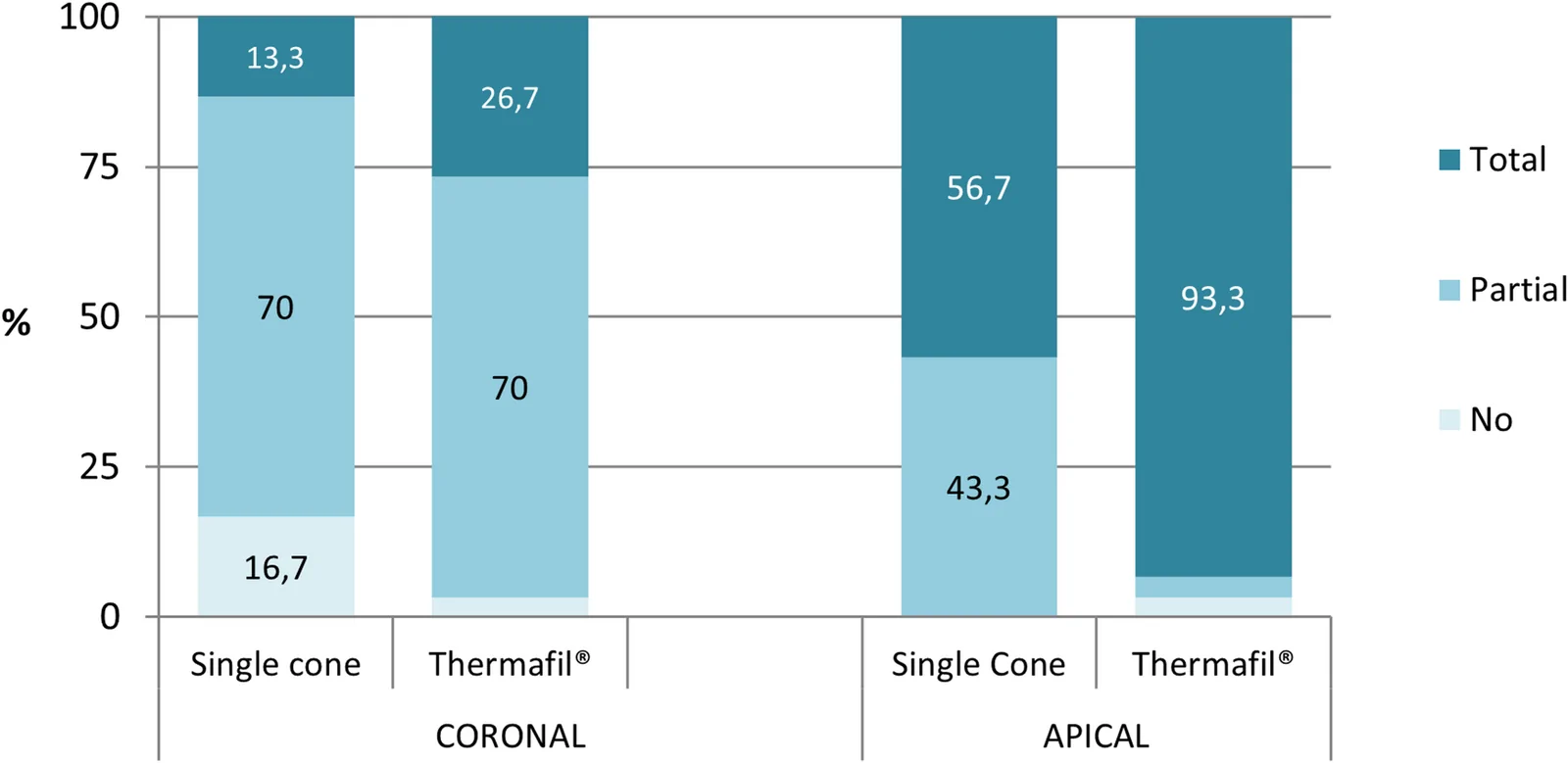
Radiographic evaluation of lateral canal obturation immediately after obturation, comparing single-cone and Thermafil^®^ techniques at coronal and apical canal levels

**Fig. 4:**
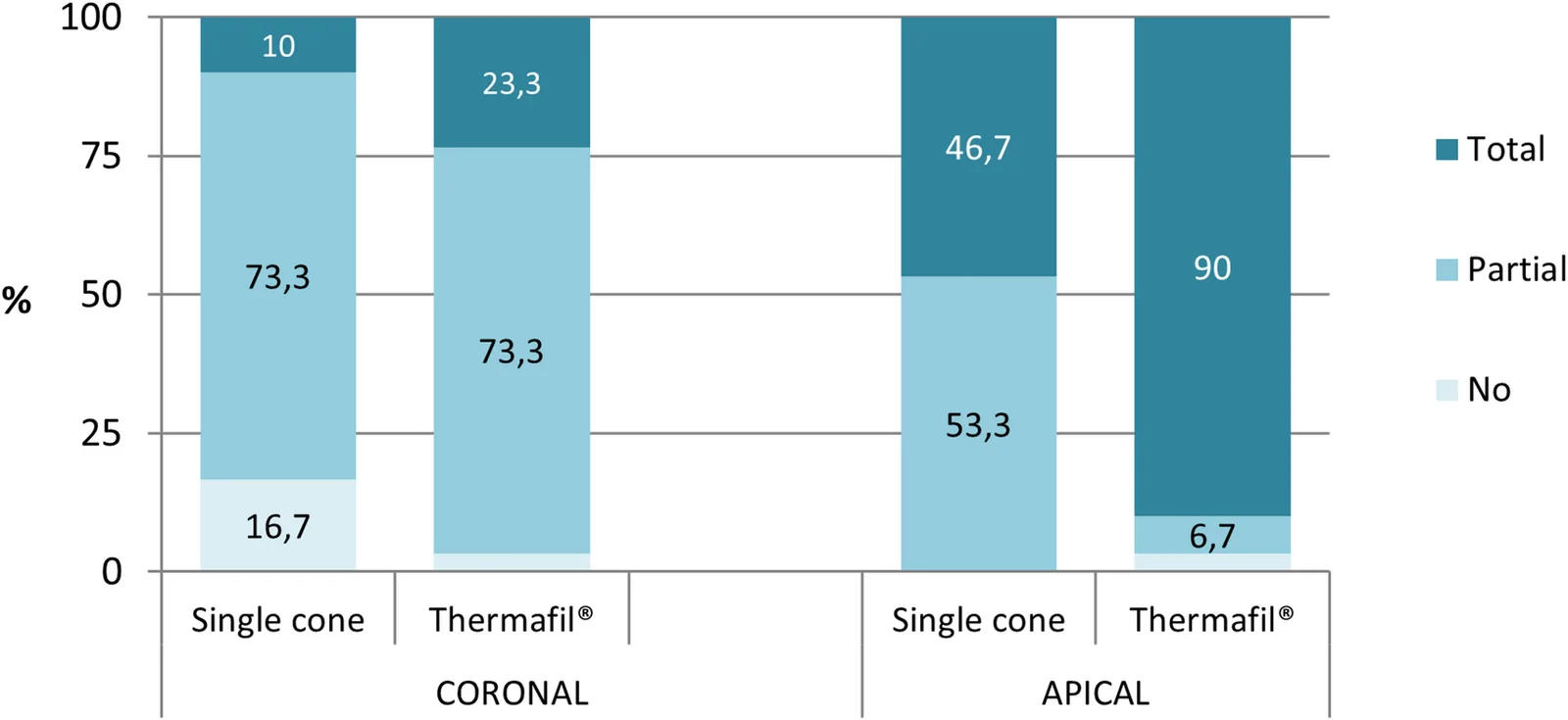
Radiographic evaluation of lateral canal obturation after complete sealer setting (4 days), comparing single-cone and Thermafil^®^ techniques at coronal and apical canal levels. No significant differences were observed compared with the immediate evaluation

A total of 120 lateral canals were analyzed, distributed according to the obturation technique used (single-cone or Thermafil^®^), the canal position (coronal or apical), and the evaluation time (immediate and at 4 days). The data are presented in detail in Figs. 3 and 4; Table 1 .

**Table 1:** Distribution of obturation material in lateral canals according to obturation technique, canal location and evaluation time

Technique	Canal level	Evaluation time	Gutta-percha only *n* (%)	Sealer only *n* (%)	Gutta-percha + sealer *n* (%)
Single_cone	Coronal	Immediate	0 (0)	15 (100)	0 (0)
Single_cone	Coronal	4 days	0 (0)	15 (100)	0 (0)
Single_cone	Apical	Immediate	0 (0)	15 (100)	0 (0)
Single_cone	Apical	4 days	0 (0)	15 (100)	0 (0)
Thermafil^®^	Coronal	Immediate	0 (0)	6 (20.7)	23 (79.3)
Thermafil^®^	Coronal	4 days	0 (0)	5 (17.2)	24 (82.8)
Thermafil^®^	Apical	Immediate	1 (3.4)	3 (10.0)	26 (86.7)
Thermafil^®^	Apical	4 days	1 (3.4)	3 (10.0)	26 (86.7)

No significant differences were observed between immediate and post-setting evaluations (McNemar test, *p* = 1.0)

### Effect of the obturation technique

Radiographs taken the day of obturation showed that at the apical lateral canals, the Thermafil^®^ technique achieved a significantly higher rate of complete obturations (93.3%) compared to the single-cone technique (56.7%) (*p* = 0.001, Fisher’s test). At the coronal level, the rates were lower for both techniques: 26.7% with Thermafil^®^ and 13.3% with the single-cone technique, with no statistically significant differences between them (*p* = 0.197, Chi-square test) (Fig. 3).

In the 4-day radiographic control, after the sealer had set, complete obturation of apical lateral canals reached 90% with Thermafil^®^ and 46.7% with the single-cone technique (*p* < 0.001). In coronal lateral canals, the rates were 23.3% and 10%, respectively, with no significant differences (*p* = 0.166) (Fig. 4). Overall, Thermafil^®^ demonstrated superior effectiveness, particularly in apical canals.

### Effect on the distribution of filling material into apical and coronal lateral canals

Regardless of the obturation technique used, apical lateral canals exhibited a higher rate of complete obturation than coronal canals. At the immediate follow-up, the overall complete obturation rate was 75% for apical canals versus 20% for coronal canals (*p* < 0.001, McNemar test). This trend was confirmed at the second evaluation, with rates of 68.3% for apical canals and 16.7% for coronal canals (*p* < 0.001) (Figs. 3 and 4).

In the analysis by technique, Thermafil^®^ showed significantly higher rates of complete obturation of the apical lateral canals (93.3% at immediate follow-up and 90% at 4 days) compared to the coronal lateral canals (26.7% and 23.3% respectively; *p* < 0.001 in both cases).

### Effect of setting time of the sealer

When comparing the results between the two radiographic evaluations, no statistically significant differences were observed, indicating that exposure to heat did not adversely affect the dimensional stability of the sealer after setting.

### Determination of the material composition within lateral canals

The photographs enable visual assessment of the type of material present within the lateral canals. In all cases treated with the single-cone technique, only sealer was observed. In contrast, in the Thermafil^®^ group, a combination of gutta-percha and sealer predominated—both coronally (79.3% at the immediate follow-up and 82.8% at 4 days) and apically (86.7% at both dates) (Table 1). The differences between techniques were statistically significant (*p* < 0.001).

Furthermore, no differences were observed between the identification of the material on both dates (*p* = 1, McNemar test).

## Discussion

Proper obturation of lateral canals is essential in endodontics, as it creates a barrier against reinfection and contributes to treatment success [18]. In the present in vitro study, the efficacy of two obturation techniques—single-cone and Thermafil^®^—was compared in the filling of standardized lateral canals, while also evaluating the influence of lateral canal position, the type of material present within the canals, and the behavior of the CeraSeal^®^ bioceramic sealer after setting.

### Influence of the obturation technique

The results suggest that Thermafil^®^ technique was more effective in filling lateral canals than the single-cone technique, especially in the apical third. This finding supports previous hypotheses and is consistent with the literature, which acknowledges the superior adaptability of thermoplastic techniques to the complex anatomy of the root canal system [10, 14, 19]. This difference may be explained by the ability of heated gutta-percha to flow and adapt three-dimensionally, allowing greater penetration into lateral canals.

In contrast, the single-cone technique showed limitations. However, the use of bioceramic sealers such as CeraSeal^®^ may partially compensate for this weakness. Its small particle size and hydrophilic behavior allow it to penetrate microstructures without the need for additional pressure [10]. Despite this, as confirmed by previous studies, obturation relying solely on sealer is not ideal in terms of long-term stability [18].

Overall, these findings support the superiority of thermoplastic techniques such as Thermafil^®^, particularly when combined with advanced materials like bioceramic sealers. However, they also highlight that even less effective techniques may benefit from the use of materials with favorable physicochemical properties.

### Influence of lateral canals position

Regardless of the technique used, the obturation rate and quality were higher in apical lateral canals than in coronal ones. This observation can be attributed to morphological differences: the cervical third of the canal has a larger diameter and an oval shape, making it more difficult for the obturation material to completely fill the area [20]. The present results are consistent with studies such as those by Loiacono et al. [20] and Somma et al. [21], who also observed a higher incidence in the coronal sres. When a single cone of gutta-percha is placed into a canal previously filled with bioceramic sealer, it generates hydraulic pressure that facilitates sealer distribution within the canal system and may be more effective in the apical third than in the coronal third (Kucuk et al., 2024). Consequently, this mechanism may result in improved obturation of apical lateral canals [13].

### Effect of the sealer setting

In the present study, no significant differences were observed between the radiographic and microscopic evaluations performed immediately after obturation and after 4 days of setting. These findings suggest that, under the conditions employed, heat-assisted did not negatively influence the short-term dimensional stability or distribution of the calcium silicate–based sealer within lateral canals. Therefore, the application of heat appears not to compromise the integrity of the root canal filling during the initial setting phase, as evaluated by radiographic and microscopic methods.

Bioceramic sealers undergo hydration reactions that are influenced by environmental factors such as moisture and temperature, both of which may affect their setting behavior and physicochemical properties [22]. Previous studies have reported that the application of heat can alter parameters such as flowability, film thickness, and setting kinetics [23]. However, these physicochemical characteristics were not directly assessed in the present study. Nevertheless, evidence from other investigations suggests that heat does not necessarily compromise the ability of certain calcium silicate–based sealers to penetrate dentinal tubules, indicating that the sealing performance may be maintained despite thermal exposure during obturation procedures [24, 25].

Therefore, although heat-related alterations reported in the literature cannot be ruled out, they were not reflected in the radiographic or microscopic outcomes evaluated in the present study. Consequently, these findings should be interpreted as evidence of short-term stability at the observational level employed, rather than as a comprehensive assessment of the sealer’s physicochemical behavior under thermal conditions.

### Obturation material composition

Photographic analysis revealed a clear association between each technique and the type of material present in the lateral canals. The single-cone technique was exclusively associated with sealer, while Thermafil^®^ was linked to the combined presence of gutta-percha and sealer. These results are consistent with several previous studies [15, 16, 17, 21], which document the ability of Thermafil^®^ and other techniques using warm gutta-percha to introduce gutta-percha into accessory canals—a capability that techniques such as the single-cone technique or cold lateral condensation cannot achieve.

Several authors observed the presence of gutta-percha in all lateral canals when the Thermafil^®^ technique was used, in contrast to the cold lateral condensation technique, where gutta-percha was not detected in any of the lateral canals [15, 17].

Similarly, it was observed that the carrier-based technique (such as Thermafil^®^) can generate a significantly greater amount of gutta-percha in all lateral canals while the cold lateral condensation technique showed a significantly higher proportion of sealer [16].

The photographs taken in this study confirmed that the technique used directly influences the distribution of obturation material and composition of the canal obturation, a key aspect for the long-term success of the treatment.

### Methodology

The methodology of this study was designed to evaluate, in a controlled and reproducible manner, the effectiveness of two obturation techniques –carrier-based and single-cone– in lateral canal filling using a bioceramic sealer (CeraSeal^®^). The use of transparent resin blocks eliminated the anatomical variability inherent to natural teeth, allowing standardized assessment of lateral canal obturation. This experimental approach is consistent with previous comparative studies that have employed simulated canal models to investigate the performance of different obturation techniques under standardized conditions.

Synthetic models offer advantages including standardized canal geometry, precise positioning of lateral canals, and enhance clear microscopic visualization. However, they also present inherent limitations, such he absence of dentinal moisture, the lack of anatomical irregularities, and thermal properties that differ from those of natural dentine. These factors may influence sealer behavior, setting dynamics, and material adaptation, and should therefore be considered when extrapolating the present findings to clinical conditions [15–17, 25].

It should also be acknowledged that the study was conducted using Thermafil^®^ training blocks. Although these models facilitated a high degree of standardization, their canal geometry was specifically designed for the Thermafil^®^ system, which may have favored the adaptation performance of the carrier-based obturation technique.

Although specimens were stored under 100% relative humidity to facilitate sealer setting, the experimental model lacked intrinsic dentinal moisture present in the natural teeth. Therefore, the hydration process and setting kinetics of calcium silicate–based sealers may differ from those occurring under clinical conditions, potentially limiting the direct extrapolating of findings to the clinical setting.

Resin exhibits thermal properties different from those of natural dentine, which may affect heat dissipation during thermoplastic obturation and, consequently, influence both gutta-percha flow and sealer behavior. Furthermore, the experimental methodology employed in this study does not permit direct evaluation of physicochemical properties, such as hydration reactions, flow characteristics, or setting kinetics following heat exposure. Therefore, the present findings should be interpreted within the context of the observational parameters assessed rather than as a comprehensive characterization of the material´s behavior under thermal conditions.

Although heat application was standardized according to the manufacturer’s instructions, the actual intracanal temperature was not measured during the obturation procedure. Consequently the potential effects of localized thermal variations on the physicochemical properties and setting behavior of the sealer remain uncertain. This limitation should be considered when interpreting the influence of heat on material performance.

Unlike studies conducted using extracted human teeth [8, 14, 20, 21], the present model does not reproduce material–dentin interaction. However, it allows isolated evaluation of material and technique behavior in a controlled environment, which was the main objective of this study.

The setting conditions (37 °C and 100% humidity for 4 days) were considered sufficient to ensure complete sealer setting. However, long-term factors that may influence material performance, such as solubility, dissolution, and aging were not evaluated in the present study.

Finally, although the use of Thermafil^®^ with a bioceramic sealer is consistent with recent studies evaluating the interaction between thermoplastic obturation techniques and calcium silicate–based sealers [10, 14, 21], the present findings may not be directly applicable to other heat-assisted system such as System B or hybrid techniques, may exhibit different behaviors [16, 17, 25]. Therefore, caution should be exercised when extrapolating these results to other thermoplastic approaches, and further studies are needed to clarify the influence of temperature, gutta-percha flow, and obturation technique on the lateral canal filling.

### Analysis methods

Regarding the analysis methods, the combined use of digital radiography and direct microscopy is consistent with previous studies employing standardized transparent resin models, which allows direct visualization and reproducible two-dimensional assessment of lateral canals filling [15, 17]. The transparency and homogeneity of resin facilitated microscopic evaluation of the filling materials without the interference typically associated with natural tooth structures.

Although advanced images techniques such as micro-computed tomography (micro-CT) provide comprehensive three-dimensional information on voids, material distribution, and unfilled areas [20, 21], their advantages are particularly relevant in studies involving natural teeth, where complex internal anatomy cannot be directly visualized. In the present model, the standardized geometry and transparent structure allowed reliable two-dimensional assessment without substantial loss of analytical precision.

Nevertheless, the absence of three-dimensional volumetric analysis represents a limitation of the study, particularly considering that micro-CT have is currently regarded as the reference method for evaluating obturation quality. Recent investigations using micro-CT have provided detailed assessment of void distribution and filling homogeneity in root canal systems obturated with calcium silicate–based sealers, underscoring the value of three-dimensional evaluation in endodontic research [26].

## Conclusion

Within the limitations of this in vitro study, heat-assisted obturation influenced the distribution and material composition of a premixed calcium silicate–based sealer within standardized lateral canals. Compared with the single-cone technique, he at application significantly improved the filling of apical lateral canals and increased the frequency of combined gutta-percha sealer fillings. In contrast, coronal lateral canals exhibited lower filling rates regardless of the obturation technique employed.

No significant radiographic or microscopic differences were observed between the immediate and post-setting evaluations, indicating that the distribution and integrity of the filling material remained stable during the setting period under the condition tested.

Therefore, the null hypothesis was rejected, as heat-assisted obturation significantly affected the distribution and material composition of the filling materials within lateral canals compared with the single-cone technique.

## Supplementary Information

Supplementary Material 1.

Supplementary Material 2.

## Data Availability

All data generated or analyzed during this study are included in this published article. Additional data are available from the corresponding author upon **reasonable request.**.
