# Supplementary material for: Influence of heat-assisted obturation on the distribution and post-setting stability of a calcium silicate–based endodontic sealer in simulated lateral canals: an in vitro study
Source: BMC Oral Health. 2026 Jul 3;26:1798. doi: 10.1186/s12903-026-09025-8 (PMC13599280; doi:10.1186/s12903-026-09025-8)
Supplement: Supplementary file 2 [file 12903_2026_9025_MOESM2_ESM.docx]

**PRILE 2021 Flowchart**

RATIONALE/JUSTIFICATION Limited evidence exists on the behavior of premixed calcium silicate sealers when combined with heat-assisted techniques for filling lateral canals.

AIM/HYPOTHESIS The study aimed to evaluate the effect of heat-assisted obturation (Thermafil) on the distribution of filling material, material composition, and post-setting stability of a premixed calcium silicate–based sealer in standardized lateral canals.

SAMPLES Methacrylate structures containing main root canals with moderate curvature and two lateral canals—one coronal and one apical

2 EXPERIMENTAL GROUPS Group 1: Single-cone (n = 30 canals / 60 lateral canals). ProTaper Next® X2 gutta-percha + CeraSeal® bioceramic sealer. Group 2: Thermafil (n = 30 canals / 60 lateral canals). Thermafil® carrier heated in Thermaprep 2® oven + CeraSeal® bioceramic sealer. VARIABLES: Independent: Obturation technique (cold vs. heat-assisted), canal level (coronal vs. apical), and time (immediate vs. 4 days). Dependent: Filling completeness (radiographic) and material composition (microscopic).

OUTCOME(S) ASSESSED, INCLUDE DEPENDENT VARIABLES AND TYPE Filling Completeness: Radiographic classification (Complete, Partial, or None). Material Composition: Microscopic identification of sealer and/or gutta-percha presence. Post-setting Stability: Comparison of filling outcomes between immediate and 4-day evaluations

METHOD USED TO ASSESS THE OUTCOME (S) AND WHO ASSESSED THE OUTCOME(S) Radiography: Digital evaluation (VistaScan®) for filling completeness. Microscopy: Visual identification (16x and 40x magnification) for material composition. Assessor: A single trained operator performed all treatments and outcome assessments to maintain standardization. * Conditioning: Outcomes verified immediately and after 4 days of controlled incubation (37 °C / 100% humidity).

RESULTS Apical Filling: Thermafil® significantly outperformed single-cone (90% vs. 46.7% complete filling, p < 0.001). Coronal Filling: Lower success rates for both techniques (p > 0.05). Composition: Single-cone resulted in sealer only; Thermafil® promoted combined gutta-percha and sealer. Stability: No significant changes occurred between immediate and 4-day evaluations, confirming dimensional stability.

CONCLUSION(S ) Heat-assisted obturation improves bioceramic sealer distribution in lateral canals without affecting post-setting stability, supporting its clinical use.

FUNDING DETAILS No financial support received for this research

CONFLICT OF INTEREST Materials provided by Meta Biomed; no financial compensation or other conflicts of interest declared

***From: Nagendrababu V, Murray PE, Ordinola-Zapata R, Peters OA, Rôças IN, Siqueira JF Jr, Priya E, Jayaraman J, Pulikkotil SJ, Camilleri J, Boutsioukis C, Rossi-Fedele G, Dummer PMH (2021) PRILE 2021 guidelines for reporting laboratory studies in Endodontology: a consensus-based development. *International Endodontic Journal* May 3. doi: 10.1111/iej.13542.** [**https://onlinelibrary.wiley.com/doi/abs/10.1111/iej.13542**](https://onlinelibrary.wiley.com/doi/abs/10.1111/iej.13542)**.**

**For further details visit: http://pride-endodonticguidelines.org/prile**
